# Real-World Outcomes of Chemoradiotherapy for Anal Squamous Cell Carcinoma: Insights from Northeast India

**DOI:** 10.1007/s13193-025-02505-3

**Published:** 2026-01-07

**Authors:** Abhinandan Das, Jyotiman Nath, Tapashi Das, Saswati Datta, Partha Sarathi Roy, Yanpothung Yanthan, Jahnabi Das, Rudrasankar Chakraborty

**Affiliations:** 1 Department of Radiation Oncology, Dr B. Borooah Cancer Institute, Guwahati, Assam India; 2 Department of Medical Oncology, Dr B. Borooah Cancer Institute, Guwahati, Assam India

**Keywords:** Anal cancer, Definitive chemoradiation, Squamous cell carcinoma, Nonmetastatic, Colostomy free survival

## Abstract

Anal squamous cell carcinoma (SCC) is a rare malignancy with rising incidence rates. While chemoradiotherapy (CCRT) has become the standard of care for nonmetastatic anal SCC, there is limited data on treatment outcomes from India, particularly the Northeastern region. This study aims to evaluate the clinical characteristics, treatment outcomes, and toxicity profiles of definitive CCRT for anal SCC at a tertiary cancer center in Northeast India. A retrospective, single-center study was conducted on 22 patients diagnosed with nonmetastatic anal SCC who received definitive CCRT between 2017 and 2021. Radiotherapy was delivered using 3D-CRT, IMRT, or VMAT, with concurrent chemotherapy comprising Mitomycin C (MMC) and 5-fluorouracil (5-FU). Acute and late toxicities were assessed, and survival outcomes were analyzed using the Kaplan-Meier method. The median age of the cohort was 54 years, with a male predominance (64%). The majority of patients had Stage III disease, and the most common symptoms were perianal pain and rectal bleeding. The median overall survival (OS) was 46 months, with 1-year, 2-year, and 3-year OS rates of 95.7%, 78.3%, and 63.6%, respectively. The 3-year locoregional control (LRC) and colostomy-free survival (CFS) rates were 57% and 60%, respectively. Patients receiving ≥ 54 Gy had significantly better survival outcomes. Common acute toxicities experienced included dermatitis, anemia, and neutropenia. Late toxicities were mild, with no grade 3 or 4 late effects observed. CCRT with 5-FU and MMC remains the gold standard for nonmetastatic anal SCC, offering favorable survival outcomes and preserving sphincter function. Advanced radiation techniques like IMRT and VMAT help minimize toxicity. These findings align with both regional and international studies, though further studies are essential to optimize radiation dose regimens and explore novel therapies to further improve patient outcomes.

## Introduction

Anal squamous cell carcinoma (SCC) is an uncommon malignancy with increasing incidence worldwide, particularly among women. In 2020, more than 50,000 new cases and 19,000 deaths were reported globally [1]. Human papillomavirus (HPV) infection is the primary etiological factor, similar to cervical cancer. Additional risk factors include immunosuppression, smoking, receptive anal intercourse, and chronic inflammatory bowel disease. Patients typically present with rectal bleeding, perianal pain, or, in advanced disease, faecal incontinence [2, 3].

Since the landmark work of Nigro et al. in 1974, combined chemoradiotherapy has become the cornerstone of treatment for anal SCC, achieving local control rates of 70–85% and 5-year OS rates of 66–92%. Additionally, CCRT is associated with improved quality of life and higher colostomy-free survival rates, leading to widespread adoption. Surgery is now reserved for patients with residual or recurrent disease [4, 5].

Evidence from India remains scarce and largely limited to single-centre reports. In Northeast India— a region with a high cancer burden—data on treatment outcomes for anal SCC are especially limited [6]. This study evaluates clinical characteristics, treatment outcomes, and toxicity profiles of patients with nonmetastatic anal SCC treated at a tertiary cancer centre.

### Methodology

This retrospective study was conducted at Dr. B. Borooah Cancer Institute, Guwahati. A total of 22 patients with nonmetastatic anal SCC treated with definitive CCRT between January 2017 and December 2021 were included. Patients with metastatic disease, previous pelvic irradiation, palliative intent treatment, or incomplete records were excluded. Institutional Ethical approval was obtained, and all data were collected from medical records and follow up communications and were anonymized.

Radiotherapy was delivered using 3D-CRT, IMRT, or VMAT. Planning CT scans were performed in treatment position. GTV, CTV, and PTV were defined using standard margins, including elective inguinal nodal irradiation. A pelvic dose of 45 Gy in 1.8-Gy fractions was followed by a 10–15 Gy boost (total 54–60 Gy).

Concurrent chemotherapy was prescribed with injection Mitomycin C (MMC) 10 mg/m^2^ on days 1 and 29, and concurrent 5FU 1000 mg/m^2^ on days 1–4 and days 29–32 or oral Capecitabine and MMC. Complete and differential blood counts, renal function tests, and liver function tests were done before commencing chemotherapy on days 1 and 28. No patient received induction or adjuvant chemotherapy.

Acute and late toxicities were assessed using CTCAE version 5.0 for acute and late toxicity criteria.

Statistical analysis was performed using SPSS version 29. The survival rate was calculated using the Kaplan-Meier method. Overall survival (OS) was measured from treatment onset to death, with censoring at the last contact for alive patients. Colostomy-free survival (CFS) was based on the date of colostomy or death. Patients who had a colostomy before chemoradiation were excluded from the CFS analysis. OS was defined as the time from diagnosis to radiotherapy initiation and death from any cause. In contrast, locoregional control (LRC) was defined as the initiation of radiotherapy to freedom from locoregional progression. Statistical significance was set at *p* value < 0.05.

#### Results

22 patients were included in the analysis, with a median age of 54 years (range: 31–92 years). The cohort was predominantly male (64%). The male (14) to female (8) ratio was 1.75. The majority of the patients had ECOG PS 1 (90.90%). Most patients (59%) had Stage III disease staged according to AJCC 8th edition. The most common presenting symptoms included pain during defecation (50%), per-anal bleeding (32%), and constipation (18%). Two patients (9.1%) were HIV-positive. Detailed HIV parameters (CD4 count, antiretroviral therapy) were not uniformly available in the retrospective records. The baseline patient and disease characteristics are reported in Table 1.

**Table 1:** Patient and disease characteristics of the entire study cohort

Characteristics		*n*	[%]
Gender	Male	14	[63.6]
	Female	8	[36.4]
Age (years)	Median	64 years	
	Range	(31–92)	
PS (ECOG)	1	20	[90.90]
	2	2	[09.10]
HIV	Positive	2	[9.10]
	Negative	20	[90.90]
Presenting symptom	Pain during defecation	11	[50]
	Per rectal bleed	7	[32]
	Constipation	4	[18]
T stage	1	0	
	2	7	[32]
	3	13	[59]
	4	2	[9]
N stage	0	10	[45.4]
	1a	8	[36.4]
	1c	4	[18.2]
Stage			
	IIA	5	[22.7]
	IIB	4	[18.2]
	IIIA	2	[9]
	IIIC	11	[50]
Histology	Squamous	22	[100]
	Non squamous	-	

Radiotherapy was administered using either 3D-CRT (32%), IMRT (32%), or VMAT (36%). The median total radiotherapy dose was 54 Gy (50.4–61 Gy). 71% of patients received concurrent 5-FU/mitomycin C chemotherapy. Other chemotherapy regimens used were capecitabine + MMC and cisplatin + 5FU. No patient received induction or adjuvant chemotherapy; all systemic treatment was given concurrently with radiotherapy according to the standard protocol (Table 2).

**Table 2:** Treatment characteristics, compliance and toxicity for the entire cohort

Characteristics		*N*	[%]
RT dose	Median	54 Gy	
	Range	50.4–61.2 Gy	
RT techniques	3DCRT	7	32
	IMRT	7	32
	VMAT	8	36
Concurrent CT	5FU + MMC	15	70
	5FU + CISPLATIN	4	18
	Others	3	[12]
OTT	Median (Range)	52 days (47–72)	
Acute toxicity	Any grade	20	90.90
	Grade 3–4		
	Anaemia	4	18
	Neutropenia	4	18
	Diarrhoea	2	9
	Dermatitis	4	18
Late toxicity	Any grade	6	27
	Grade 3–4	Nil	Nil

#### Survival and Toxicity

**Fig. 1:**
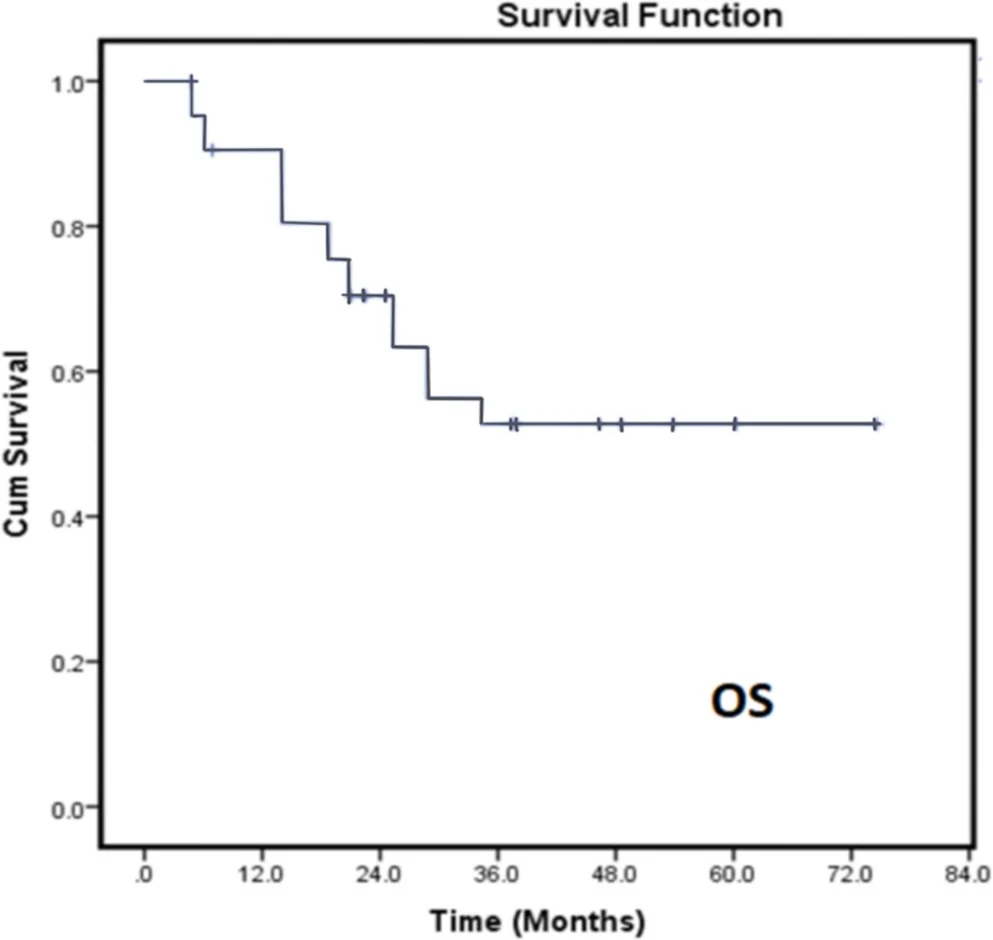
Kaplan–Meier curve illustrating overall survival

The median follow-up of the patient cohort was 48 months (6–96 months). At six months, complete response was seen in 15 patients (68%). Two patients had distant metastases, 2 had progressive disease, and one had expired. The median overall survival (OS) for the cohort was 46 months. The one year, two year and three year OS rates were 95.7%, 78.3% and 63.63% (Fig. 1), respectively. The 3-year LRC was 57% for the whole cohort. Younger patients with early T-stage and node-negative disease had better LRC.

**Fig. 2:**
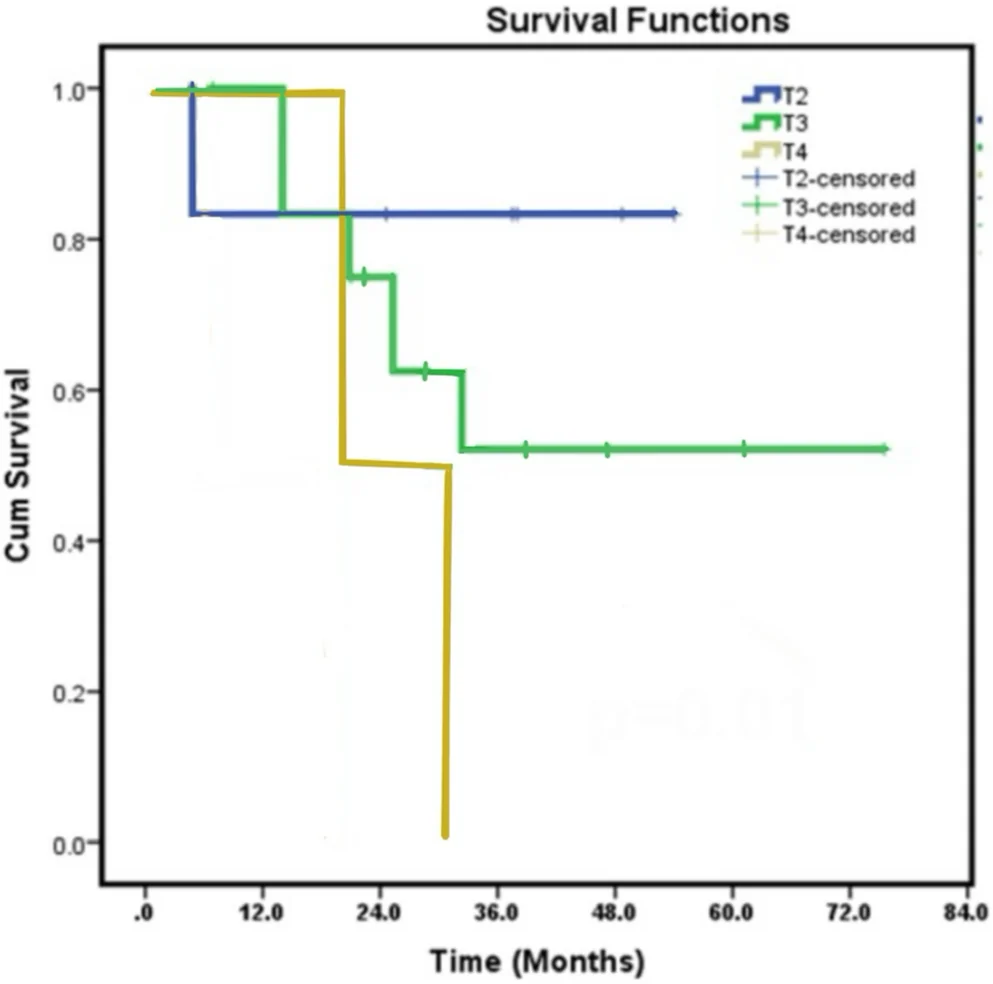
Comparison of survival among different T-stages

**Fig. 3:**
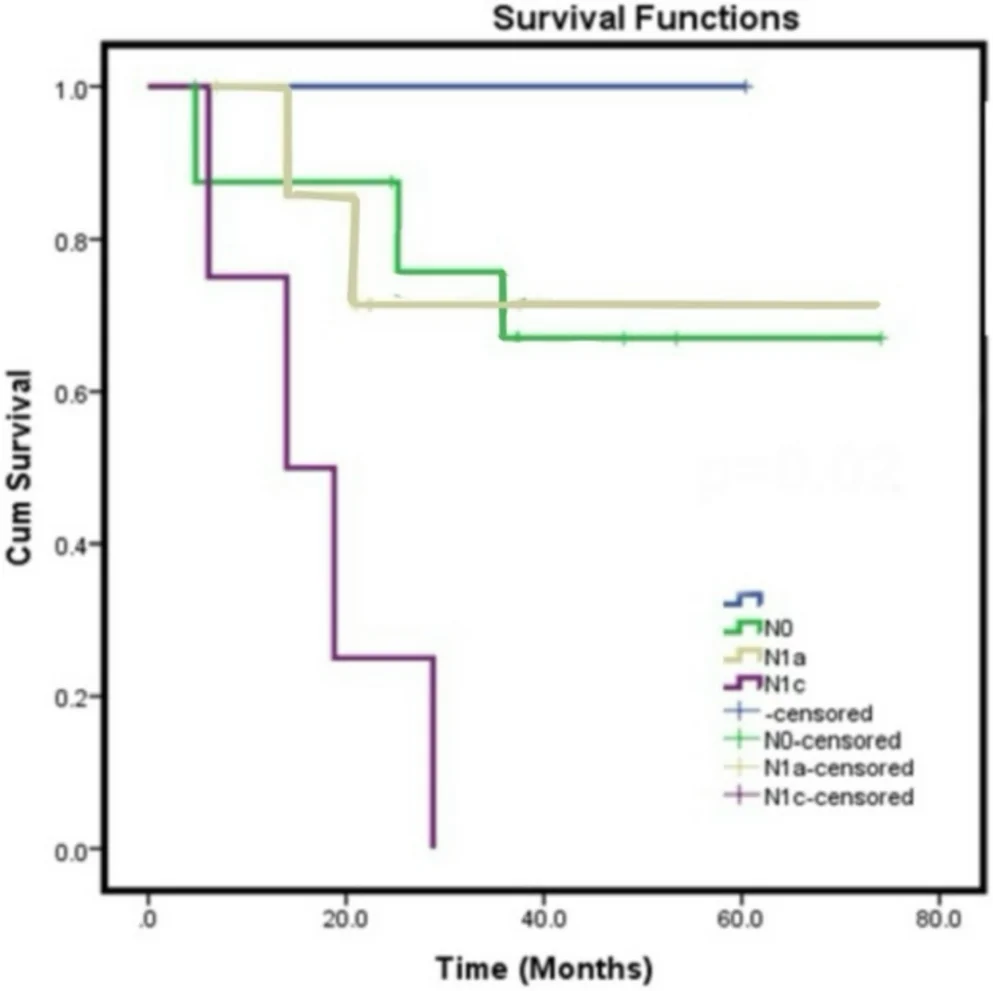
Comparison of survival among different N stages

**Fig. 4:**
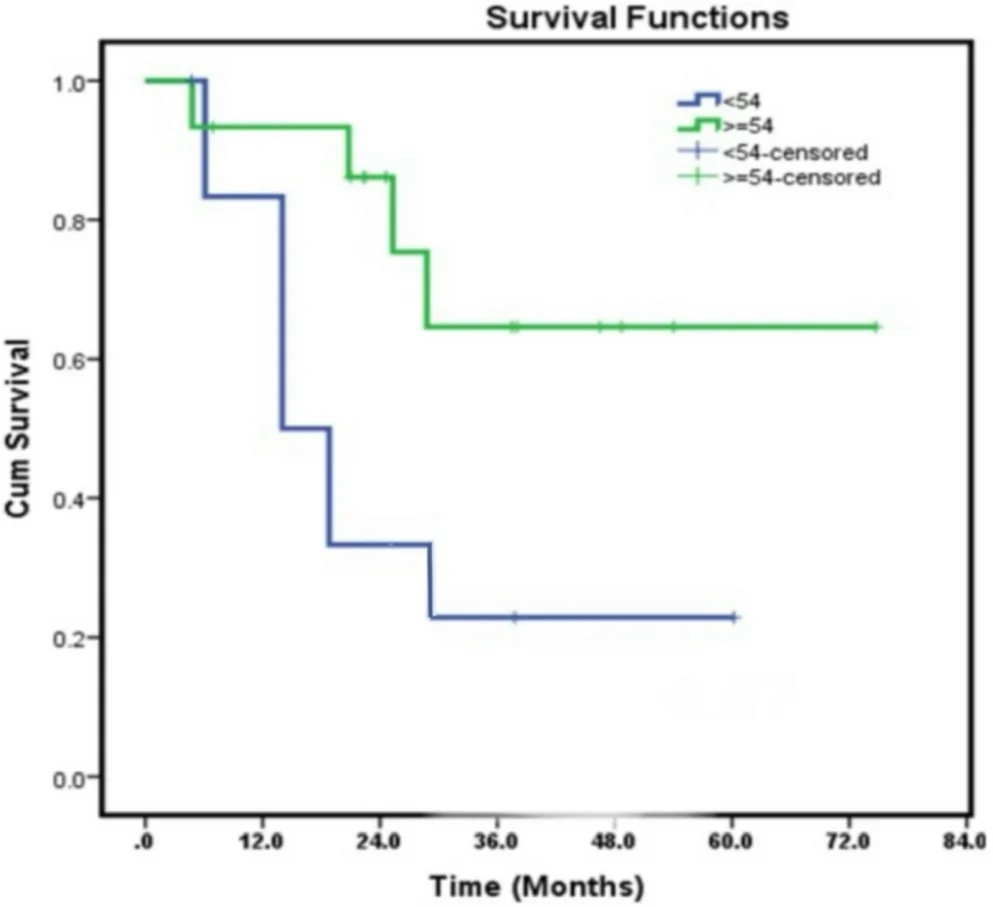
Comparison of Survival among different RT doses

At the time of the last follow-up, nine patients were alive. When stratified by disease stage, patients with Stage IIIC disease had a median survival of 29 months, while those with Stage IIB disease had a median survival of 67 months. At 36 months, survival rates for male patients were 58% compared to 75% in females. Node-negative (85.7%) and T2 (80%) disease had better 3 years survival compared to node-positive (50%) and higher T-stage disease (53%) (Figs. 2 and 3). 3-year OS for patients receiving ≥ 54 Gy were 73.33%, compared to 33.3% for those receiving lower doses (Fig. 4). Table 3 depicts univariate analysis of various tumour characteristic in relation to overall Survival.

**Table 3:** Univariate analysis of various tumour characteristic in relation to overall survival

	3-year OS 63.63%				
Prognostic Factors		Number (n)	%	3 Yrs OS	*p* value
Age	< 60	9(7)	41%	77.78%	0.2513
	> 60	13(7)	59%	53.8%	
Sex					0.65
	Male	14	63.6	57.84%	
	Female	8	36.4	75%	
T Stage	T2	7	31.8	85.7%	0.082
	T3	13	59.1	61.53%	
	T4	2	9.1	0	
N stage	N0	10	45.45	80%	0.0135
	N1a	8	36.36	75%	
	N1b	-	-	-	
	N1c	4	18.18	0.00%	
Stage	IIA	5	22.72	80%	
	IIB	4	18.18	75.00%	
	IIIA	2	9.09	50%	
	IIIC	11	50	45.45%	
RT Dose	< 54 Gy	6	27.27	33.30%	0.15
	>= 54 Gy	15	72.72	73.33%	
RT Duration	< 50 days	11	50	72.72%	0.66
	> 50 days	11	50	54.54%

The 3-year CFS rate was 60%. Two patients had undergone pre-treatment APR and were excluded from the CFS analysis. Until the end of the follow-up period, four patients had undergone colostomy due to LRR or persistent disease. The 3-year CSF rate was better in T2 (85.7%) and N0 (77.77%) diseases compared to higher T stage (46%) and node-positive (45.45%) diseases.

Table 2 summarises the acute and late treatment toxicities. Four patients (18%) required temporary interruptions in treatment due to skin reactions, and two patients required hospital stay due to diarrhoea, but all patients completed the prescribed therapy (Table 2). Toxicity data revealed that all of the patients experienced at least grade 1 toxicity, the most common being grade 1 dermatitis. Grade > 3 acute toxicities included skin reactions 4 (18%), anaemia 4(18%), and diarrhoea 2(9%). Six patients (27%) experienced grade > 3 neutropenia, managed with granulocyte colony-stimulating factors. No treatment-related deaths were reported. Clinically significant (≥ Grade 2–3) toxicities were more frequent in the 3D-CRT group compared to IMRT/VMAT, particularly for ≥ G3 dermatitis and neutropenia. Advanced techniques demonstrated a lower incidence of severe toxicity, consistent with their better dose conformity and organ-at-risk sparing.

In terms of late toxicity, there were no grade 3 or 4 late toxicities in any patient. Grade 2 late toxicities included proctitis in three, skin fibrosis in two and vaginal stenosis in one patient.

Clinically significant (≥ Grade 2–3) toxicities were more frequent in the 3D-CRT group compared to IMRT/VMAT, particularly for ≥ G3 dermatitis and neutropenia. Advanced techniques demonstrated a lower incidence of severe toxicity, consistent with their better dose conformity and organ-at-risk sparing (Table 4).

**Table 4:** Comparison of significant toxicities (≥ Grade 2) between 3D-CRT and IMRT/VMAT

Toxicity	3D-CRT (*n* = 7)	IMRT/VMAT (*n* = 15)	Total (*n* = 22)
**Acute Toxicities (≥ Grade 3)**			
Dermatitis	2 (29%)	2 (13%)	4 (18%)
Anaemia	2 (29%)	2 (13%)	4 (18%)
Neutropenia	3 (43%)	3 (20%)	6 (27%)
Diarrhoea	1 (14%)	1 (7%)	2 (9%)
Treatment Interruption	2 (29%)	2 (13%)	4 (18%)
Hospitalisation for Diarrhoea	1 (14%)	1 (7%)	2 (9%)
**Late Toxicities (≥ Grade 2)**			
Proctitis	1 (14%)	2 (13%)	3 (14%)
Skin Fibrosis	1 (14%)	1 (7%)	2 (9%)
Vaginal Stenosis	0	1 (7%)	1 (5%)
Late Toxicity	0	0	0

## Discussion

The primary goal in treating localized anal cancer is to achieve locoregional control while preserving organ function.

Before the 1980 s, the standard treatment for localized anal cancer was surgical resection, often involving abdominoperineal resection (APR), which necessitated a permanent colostomy. The surgical series showed that the 5-year overall survival rate following APR ranged from 40% to 70%. Still, the procedure was associated with significant morbidity, including loss of sphincter function and high rates of sexual and urinary dysfunction [7].

Dr. Norman Nigro and colleagues introduced neoadjuvant chemoradiotherapy before APR, combining 5-fluorouracil (5-FU) and mitomycin C (MMC). This regimen enabled some patients to achieve a complete pathological response, eliminating the need for surgery [8].

The UKCCCR/ACT1 and EORTC trials in the late 1990 s demonstrated the superiority of chemoradiotherapy over radiotherapy alone in achieving better local disease control, colostomy-free survival, and disease-specific survival. Both trials used 45 Gy of whole pelvic radiotherapy followed by boost and concurrent 5-FU and MMC, showing similar outcomes [9, 10].

However, the inclusion of MMC in the Nigro protocol contributes to significant adverse effects, including leukopenia, thrombocytopenia, nephrotoxicity, and pulmonary toxicity. Alternative chemotherapy agents, such as cisplatin (CIS) and capecitabine, have been evaluated to reduce side effects. Still, the ACT II trial showed that cisplatin-based regimens did not outperform standard treatment. Additionally, cisplatin requires intravenous hydration and anti-emetic therapy, making 5-FU with MMC the standard of care [11].

Our cohort’s 3-year OS rate of 63.6% is comparable to major trials. The UKCCCR/ACT1 trial reported a 3-year OS of approximately 60%, while RTOG 9811 showed higher survival (84%) in patients treated with 5-FU and MMC. Similarly, the EORTC and ACT II trials confirmed the effectiveness of 5-FU/MMC-based chemoradiotherapy. In our study, the 1-, 2-, and 3-year OS rates were 95.7%, 78.3%, and 63.6%, respectively. The decline from 1 to 3 years is largely due to late locoregional and distant recurrences occurring after the first year, and the small cohort size further accentuates percentage shifts over time.

Our findings are also consistent with these studies, which demonstrate that chemoradiotherapy offers survival rates on par with surgical interventions while reducing the morbidity associated with colostomy and organ dysfunction.

Our data is also comparable to the regional data from India, albeit with some variability in outcomes. The study by Bharti et al. reported a 3-year OS of 54% in patients with anal SCC treated with CCRT [12, 13]. In contrast, Das et al. demonstrated a higher 3-year OS of 81% in a cohort treated with concurrent chemotherapy and intensity-modulated radiation therapy (IMRT). Our study’s survival rates fall between these two findings, indicating that while CCRT is highly effective, patient outcomes may vary based on individual factors such as differences in patient populations, tumour stages, institutional protocols., disease stage, radiotherapy dose, and chemotherapy regimen.

The relationship between radiation dose and survival was another key finding in our study. Patients who received ≥ 54 Gy had a significantly better 3-year OS rate (73.33%) compared to those who received lower doses (33.3%). This result is consistent with findings from other studies, such as the RTOG 92 − 08 trial, which suggested that higher radiation doses can improve locoregional control [14]. As all patients received the same concurrent chemotherapy regimen, the survival advantage seen in the ≥ 54 Gy group is likely driven by the higher radiation dose. Nevertheless, this association cannot be definitively established due to the small sample size and retrospective design.

Six patients received < 54 Gy—three treated under earlier departmental protocols recommending 50.4 Gy for selected early-stage cases, and three due to treatment interruptions from patient logistics or machine downtime. Patients receiving ≥ 54 Gy showed better overall survival with only a slight increase in higher-grade dermatitis, though this was not statistically significant given the small cohort.

The role of radiotherapy dose escalation has been explored in several studies. The RTOG 92 − 08 trial, which delivered a total dose of 59.4 Gy in 1.8 Gy fractions with a 2-week treatment break, resulted in a higher colostomy rate of 30%, compared to 9% in RTOG 87 − 04, where patients received 45 Gy without a treatment break. In addition, larger tumour sizes (> 5 cm) were associated with worse 5-year disease-free and overall survival rates, as shown in RTOG 98 − 11 [15].

The UNICANCER ACCORD-3 trial was designed to determine whether dose escalation of the radiation boost or two cycles of Induction chemotherapy (ICT) before concomitant RCT improve colostomy-free survival (CFS). However, using CFS as their primary endpoint, they did not find an advantage for either ICT or HD radiation boost in LAACC [16].

While dose escalation may improve local control, it is associated with an increased risk of faecal incontinence and rectal complications, such as stricture and stenosis. For example, a European study found that one-third of patients treated with 56 Gy developed faecal incontinence. Therefore, carefully balancing dose escalation with potential side effects is crucial [17].

One of the key advantages of chemoradiotherapy over surgery is its ability to preserve sphincter function and avoid permanent colostomy. In our study, the 3-year CFS rate was 60%. Two patients who had received pre-treatment APR were excluded from the analysis. By the end of the follow-up, four patients had to undergo colostomy due to local recurrence (LRR) or persistent disease. The 3-year CFS rate was higher in patients with T2 (85.7%) and N0 (77.7%) disease compared to those with higher T stages (46%) or positive lymph nodes (45.45%).

In our study, all patients experienced at least grade 1 toxicity, with dermatitis being the most common side effect. More severe acute toxicities, such as grade 3 anaemia, neutropenia, and diarrhoea, occurred in a minority of patients (18%, 18%, and 9%, respectively). These toxicities were managed with supportive care, including colony-stimulating factors for neutropenia and symptomatic treatment for diarrhoea. No treatment-related deaths were reported, which suggests that the regimen was generally well tolerated despite the occurrence of toxicities.

Our toxicity data align with those reported in the RTOG 9811 and UKCCCR/ACT1 trials, where acute toxicities, including skin reactions, neutropenia, and gastrointestinal issues, were common but manageable with appropriate care. Late toxicities, such as proctitis, skin fibrosis, and vaginal stenosis, were mild in our cohort, with no grade 3 or 4 late effects observed. This is a favourable finding, as long-term sequelae of pelvic radiation can be debilitating, particularly proctitis and skin fibrosis, which significantly impact quality of life. These findings are consistent with other studies, including the RTOG 9811 trial, which reported similar late effects, albeit in a smaller proportion of patients.

In our study, the absence of grade 3 or 4 late toxicities is due to the use of conformal radiotherapy techniques such as IMRT and VMAT allowing for better sparing of critical organs, such as the bowel, bladder, and sexual organs, thereby reducing the risk of severe long-term complications. It reinforces the importance of advanced radiotherapy in reducing late toxicities, further supporting its role in the treatment of anal SCC.

Chemoradiotherapy is an effective initial treatment for anal cancer, though local progression is the most frequent form of recurrence. Salvage surgery can offer favourable long-term outcomes, despite the increased risk of complications due to the effects of prior chemoradiotherapy on wound healing. In this study, patients with local recurrence either underwent salvage surgery or palliative chemotherapy. Re-irradiation in recurrent setting may be considered. Osborne et al. reported a 60% three-year overall survival with hyper-fractionated re-irradiation, with manageable late adverse effects [18]. 

With the availability and widespread use of precise diagnostic imaging and in the era of Intensity-Modulated Radiation Therapy (IMRT), both dose de-escalation and dose escalation continue to be investigated, for tailoring out treatment strategy for patients with different stages of disease. The PLATO protocol consists of three trials (ACT3, ACT4, and ACT5) designed to optimize chemoradiotherapy dosing for anal cancer patients based on their risk levels. ACT3 is a phase II trial comparing surgery alone for early-stage tumors with margins > 1 mm and low-dose radiotherapy with chemotherapy for tumors with close margins (≤ 1 mm). ACT4 is a randomized phase II trial comparing standard-dose (50.4 Gy) with reduced-dose (41.4 Gy) chemoradiotherapy in intermediate-risk patients, aiming to minimize side effects while maintaining efficacy. ACT5 is a randomized seamless pilot/phase II/III trial comparing standard-dose (53.2 Gy) with higher doses (58.8 Gy and 61.6 Gy) in patients with locally advanced anal cancer, seeking to reduce recurrence rates without significantly increasing side effects [19].

Our study is one of the first studies from Northeast India that has reported the outcomes and prognostic factors for squamous cell carcinoma of the anal canal and confirms the findings reported in the literature.

Several limitations of our study should be acknowledged. The retrospective, non-randomized design and small sample size limit the generalizability of our findings. Although the study provides valuable insights into the treatment of anal SCC in a resource-limited setting, larger prospective studies with longer follow-up are needed to confirm our results. Additionally, while the survival outcomes in our cohort are comparable to those observed in international studies, there is a need for further research to assess the long-term impact of chemoradiotherapy on patient quality of life and late toxicities and also regarding dose de-escalation and escalation as per risk categories.

Future studies should also explore the role of novel therapies, such as immunotherapy and targeted therapies, in the treatment of anal SCC. These therapies may offer additional treatment options for patients with advanced or recurrent disease and may help improve survival outcomes while minimizing toxicity.

### Conclusion

Definitive chemoradiotherapy with 5-FU and MMC remains the gold standard for nonmetastatic anal SCC, offering satisfactory survival outcomes while preserving sphincter function. Our findings align with regional and international studies, highlighting its effectiveness. Although toxicity is a concern, advanced techniques like IMRT and VMAT show promise in reducing side effects. Ongoing research, including novel therapies and optimized regimens, is crucial for improving outcomes. In conclusion, personalized treatment strategies, such as dose modifications based on disease stage, should be explored to enhance efficacy and minimize toxicity, ultimately improving patient outcomes.
